# Absence of Sex Differential Plasticity to Light Availability during Seed Maturation in *Geranium sylvaticum*

**DOI:** 10.1371/journal.pone.0118981

**Published:** 2015-03-04

**Authors:** Sandra Varga, Ester Laaksonen, Pirkko Siikamäki, Minna-Maarit Kytöviita

**Affiliations:** 1 Department of Biological and Environmental Science, University of Jyväskylä, P.O. Box 35, 40014, Jyväskylä, Finland; 2 Department of Biology, University of Oulu, P.O. Box 3000, 90014, Oulu, Finland; 3 Metsähallitus, P.O. Box 26, 93601, Kuusamo, Finland; Helmholtz Centre for Environmental Research - UFZ, GERMANY

## Abstract

Sex-differential plasticity (SDP) hypothesis suggests that since hermaphrodites gain fitness through both pollen and seed production they may have evolved a higher degree of plasticity in their reproductive strategy compared to females which achieve fitness only through seed production. SDP may explain the difference in seed production observed between sexes in gynodioecious species in response to resource (nutrients or water) availability. In harsh environments, hermaphrodites decrease seed production whereas females keep it relatively similar regardless of the environmental conditions. Light availability can be also a limiting resource and thus could theoretically affect differently female and hermaphrodite seed output even though this ecological factor has been largely overlooked. We tested whether the two sexes in the gynodioecious species *Geranium sylvaticum* differ in their tolerance to light limitation during seed maturation in the field. We used a fully factorial block experiment exposing female and hermaphrodite plants to two different light environments (control and shade) after their peak flowering period. Specifically, we measured fruit and seed production in response to decreased light availability and compared it between the sexes. Shading reduced the number of fruits and seeds produced, but the decrease was similar between the sexes. Furthermore, shading delayed seed production by three days in both sexes, but did not affect seed mass, seed P content, or the probability of re-flowering the following year. Our results give no evidence for reproductive SDP in response to light during seed maturation.

## Introduction

The most widespread breeding system among sexually dimorphic plants is gynodioecy [1], where populations contain hermaphrodite and female individuals (see e.g. [2]). Gynodioecy emerges when a female mutant invades and effectively spreads within a hermaphroditic population (see [3], [4] and [5] for reviews on the topic). One necessary condition for female maintenance in gynodioecious populations is the existence of female advantage: females must compensate for not fathering offspring like hermaphrodites do and should therefore produce more seeds or better seeds relative to those of hermaphrodites [6],[7]. The exact amount of compensation needed depends on the sex determination system [6],[8], [9], but once females are in the population, the male function in the hermaphrodites becomes more pronounced, because they fertilize the ovules of females as well as the ovules of hermaphrodites [7], [10]. This situation has been hypothesized to lead into full separation of female and male sexes in different individuals (i.e. dioecy).

The evolutionary transitions from hermaphroditism to dioecy have received extensive attention from evolutionary biologists for decades. Gynodioecy-dioecy pathway has received the most attention among the possible routes (reviewed in [5]). Besides the relevance of the identity of the genetic mechanisms underlying this process, several ecological factors have been identified to greatly impact the evolution of the gynodioecy-dioecy pathway: pollen limitation, selfing and inbreeding depression, antagonistic and mutualistic interactions and sex allocation plasticity (see [5] and references therein). Sex allocation plasticity differences between females and hermaphrodites were hypothesized by Delph [11], [12] to explain the variation in population sex ratios across resource gradients. Delph [11] suggested that since hermaphrodites gain fitness through both pollen and seeds, they may have evolved a higher degree of plasticity in their reproductive strategy than females (sex-differential plasticity hypothesis or SDP). SDP hypothesis implies that hermaphrodites decrease seed production under harsh conditions and instead allocate resources to pollen production which is less costly and usually precedes seed production (reviewed in [13], [14]). Opposite to this, as females achieve their fitness only through seed production, females may have evolved to keep seed production relatively consistent regardless of the environmental conditions [11].

The SDP hypothesis has received empirical support for several ecological factors including habitat nutrient or water availability, pollination limitation, herbivory incidence and inbreeding depression (see [15] and references there). These studies show that hermaphrodites produce fewer seeds in dry or nutrient-poor environments whilst seed production in females remains rather stable. As far as we are aware of, the role of light availability on SDP has been investigated only in one species [16], [17] even though light availability might be an important ecological factor controlling female invasion and maintenance in gynodioecious species. In the present study we investigated whether *Geranium sylvaticum* plants show SDP in response to light availability by shading plants in the field. Light is particularly important ecological factor for this species as the plants grow in both high light (meadows and road verges that receive full sky light conditions) and low light (under forest canopy) conditions. Specifically, we tested whether the sexes differ in their tolerance to light limitation during seed maturation, a period when plants may receive less light due to the natural closing of the canopy or as a result of self-shading. According to the SDP hypothesis, we predicted that hermaphrodites would show a more pronounced seed reduction in response to shade than females. Moreover, cost of reproduction has been demonstrated in several perennial species as increased mortality and or reduced flowering probability, growth, and reproductive effort the following year, including *G*. *sylvaticum* [18]. Light availability affects plant allocation priorities and individuals under low light may allocate more resources into clonal reproduction and vegetative parts in comparison to plants under bright light [19]. However, according to life history theory, selection should favor higher reproductive effort in environments of higher adult mortality, where the probability of surviving to the next reproductive season is lower [20]. In this study, we also measured the effect of shading on plant survival and estimated the effect of reproductive output on survival and reproductive effort the following year using plants that were shaded the first year, but grown under non-shaded conditions the second year. We predicted that the seed production during the year of shading would correlate with the seed production the year after the shading. For the shaded surviving individuals, we expected higher seed production the year after shading (i.e. the plants would compensate for the decreased seed production the previous year) and for control individuals we expected a decrease in seed production the following year if costs of reproduction occur.

## Materials and Methods

### Study species

*Geranium sylvaticum* L. (Geraniaceae) is a self-compatible [21], rhizomatous, perennial plant. It has Eurasian distribution [22]. In Fennoscandia, it is common in herb-rich forests, in meadows and along roads. One individual consists of one rhizome with one to several floral shoots and several rosette leaves. In the study area, *G*. *sylvaticum* starts flowering in mid-June and the plants are pollinated by bumblebees, syrphid flies and other dipterans [23]. After flowers are pollinated, the style elongates and the five locules become visible by naked eye. Fruits normally contain up to five seeds encased individually by a cup-like structure. Seeds are forcibly ejected when mature and the capsule parts remain intact in the plant long after the seed has been ejected. Most populations are gynodioecious [21], [24], containing both female and hermaphrodite individuals in the same population. The exact mechanism of sex determination in this species is currently unknown, but it has been suggested to be determined both by cytoplasmic male sterility factors and nuclear restorers [25]. Female frequency has been shown to vary between 0.4 to 27.2% among populations in Finland [24].

### Study site

**Fig 1:**
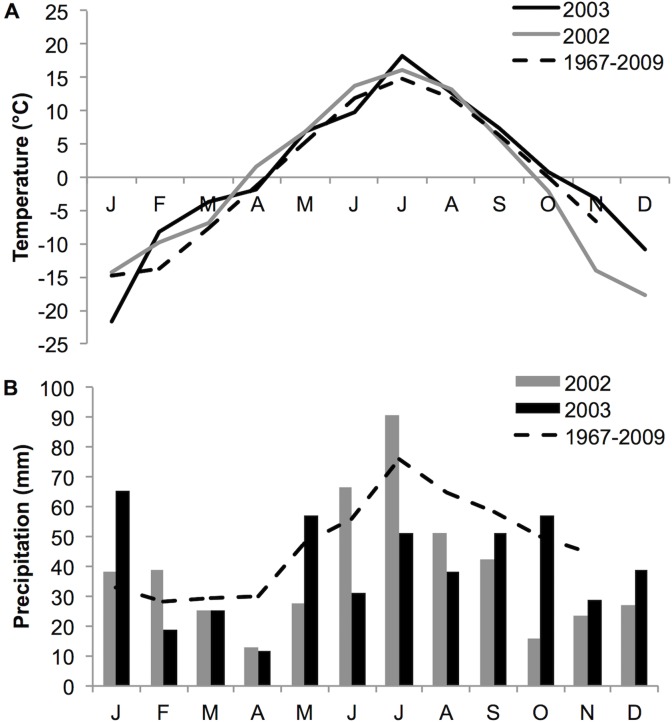
(A) Monthly averaged mean temperature (°C) and (B) monthly accumulated precipitation (mm) measured in Oulanka research station during the years of the study. The mean values calculated from 1967 to 2009 are indicated with a broken line.

This study was conducted during summer 2002 and 2003 in Liikasenvaara (66°22’N, 29°30’E), located 70 km north of Kuusamo, NE Finland, in a 0.25 ha abandoned field. No specific permissions were required to carry out the work which did not involve endangered or protected species. The field was under agricultural use (mainly hay production) until it was abandoned in 1990. The soil is mildly acidic (pH 5.5) and the nutrient levels were 1.41 μg P g^-1^, 8.95 μg K g^-1^ and 8% of soil organic matter content (dry weight) as measured in [26]. *Geranium sylvaticum* was one of the most abundant plants in the meadow and the female frequency was 24% as measured in [27]. Mean precipitation and temperature measured in the Oulanka research station 10 km W from the study site for 2002 and 2003 are given in Fig. 1.

### Experimental design

We performed a fully factorial block experiment with two factors: plant sex (female, hermaphrodite) and light availability (control, shaded). In 2002, we selected 80 plants naturally growing in the field that were included in 20 randomly located blocks throughout the field. Each block contained two females and two hermaphrodites of similar size and with a similar amount of flowering shoots, with a distance between plants within a block less than two meters.

In the beginning of the flowering season the plants were sexed and marked and the number of floral shoots and flowering structures (buds+flowers) were noted. Half of the plants were assigned to the shade treatment (referred to as Shaded plants hereafter) whilst the other half remained as controls (referred to as Control plants hereafter). Thus, each block comprised one control (non-shaded) female, one control (non-shaded) hermaphrodite, one shaded female and one shaded hermaphrodite plant of similar size. Shading frames of 80 x 80 x 80 cm covered with black woven polypropylene strawberry fabric (MyPex, UK) where placed on top of each shaded plant on 29^th^ June when most of the flowers were already pollinated until the end of the growing season (8^th^ August). The fabric covered also the top 20 cm of the vertical sides. The upper parts of the shaded plants received 10% of the available light relative to control plants and rosette leaves received between 20 and 30% of the light relative to control plants as measured with Li-Cor 6400 photosynthesis analyzer (Lincoln, NE, USA).

### Reproductive measurements

The plants were scored again for the number of flowering structures (buds+flowers) and fruits on 20^th^ July 2002. During the growing season in 2002, seeds were collected on a daily basis from 18^th^ July to 1^st^ August and on a final collection on 8^th^ August. Accumulation curves for seed production were drawn to check for differences in seed production due to plant sex and or the experimental treatment. At the end of the fruiting period, the number of fruits and seeds were counted and the shading frames were removed forevermore. The experimental plants were marked and thus we were able to locate them the following year (see below). Seed production was calculated by counting all seeds present in the fruits. In cases where the seeds had been already ejected, the number of seed scars on the base of the style was counted. Fruit set was calculated as the proportion of flowers that developed into a fruit.

Seed mass was weighed from healthy, undamaged seeds after drying them at room temperature for two days. P content of the seeds was measured from ten randomly chosen plants from each sex and light treatment (N = 40 plants) as an estimate of seed quality. Seed P content was analyzed following the procedure described in [28]. Dried and milled seeds were acid digested using the Paar001H program in the Paar Physica multiwave sample preparation system (Perkin Elmer). P concentration was measured as absorbance at 882 nm (UV-160A Shimadzu, Duisburg, Germany).

In the following year 2003, plant survival and whether the plants were reflowering or not were recorded. No experimental treatment was applied to the plants year 2003. Seed production was estimated at the end of the flowering season as described for the previous year.

### Statistical analyses

All data analyses were conducted with R (v.3.0.1; R Development Core Team, 2013). Generalized linear mixed effect models (GLMER, libraries ‘lme4’ and ‘MASS’) with negative binomial distributions were fitted to count data (number of shoots, number of floral structures) to correct for overdispersion and binomial distributions to proportional data (fruit set) and plant survival. Data on seed production in 2002 and 2003 were analyzed with Hurdle count models (library ‘pscl’) to account and model the amount of zeroes in the data, fitting binomial zero distributions and negative binomial count distributions and using Akaike’s information criteria (AIC) for selecting the best final models which are presented in the manuscript (see full models in S1 Table). The intercept was set in all cases as Control Females. Finally, the effects of plant sex and light treatment on seed mass, seed P content and date when plants had produced 50% of their seeds were investigated with two-way analysis of variance. In all models, plant sex, light treatment and their interaction were treated as the fixed component and block was used as the random component. For the data on number of floral shoots and floral structures, only the effect of sex and block were tested as the experimental treatment had not started yet. The number of floral shoots was included as a covariate to account for potential differences in plant size in all models only for variables measured in 2002 (as we did not record the number of floral shoots in 2003) after centering them to the mean to facilitate interpretation of model estimates.

## Results

**Fig 2:**
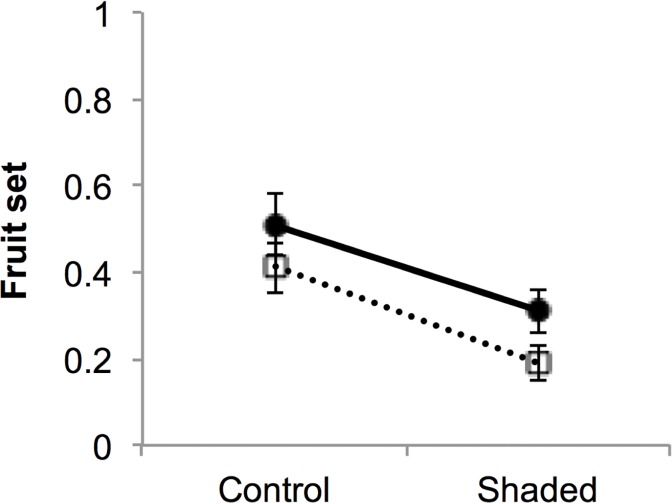
Fruit set in female (●) and hermaphrodite (□) control and shaded *Geranium sylvaticum* plants. Mean ± 1SE are indicated (N = 80 plants).

We chose plants with similar size within each block. Consequently, at the beginning of the experiment when the shading frames were placed, the sexes did not differ in the amount of floral shoots (2.2 ± 1.1 vs. 2.0 ± 1.2 in females and hermaphrodites respectively) or the amount of floral structures (42.3 ± 1.1 vs. 46.9 ± 1.0; Table 1). The shading treatment affected fruit set sex-specifically (Table 1, Fig. 2). As indicated by the model estimates, fruit set was reduced by the shading treatment in both sexes and females had significantly higher fruit set than hermaphrodites (Table 1, Fig. 2).

**Table 1:** Statistical results from the generalized linear mixed effects models.

Dependent variable		Estimate	SE	t/z value	**P value**
**Number of shoots**	Intercept	0.770	0.120	6.397	<0.001
	Hermaphrodite	-0.058	0.150	-0.385	0.700
**Number of floral structures**	Intercept	3.746	0.061	61.47	<0.001
	Hermaphrodite	0.102	0.083	1.23	0.220
	Shoots (centered)	0.296	0.035	8.48	<0.001
**Fruit set**	Intercept	0.743	0.256	2.899	0.004
	Shoots (centered)	0.077	0.031	2.524	0.012
	Shade	-1.413	0.132	-10.728	<0.001
	Hermaphrodite	-0.831	0.133	-6.267	<0.001
	Shade x Hermaphrodite	-0.321	0.186	-1.727	0.084

In all models, block was included as a random effect.

**Fig 3:**
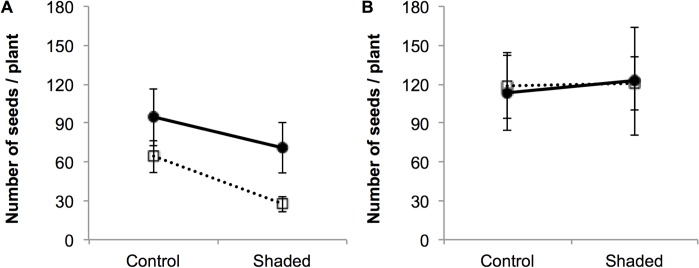
Number of seeds produced in female (●) and hermaphrodite (□) control and shaded *Geranium sylvaticum* plants that set seeds in (A) 2002 and (B) 2003. Mean ± 1SE are indicated (N = 64 plants and N = 53 plants for 2002 and 2003 respectively). In (B), Control and Shade refer to the treatments given in 2002 as no experimental treatment was applied in 2003.

Sixteen plants failed completely to produce any seed. Results from the Hurdle model indicate that fruiting probability was not related to the sex of the plant or the light treatment (Table 2). However, among the plants that did produce some seeds, differences due to shade treatment and sex were detected: shaded plants produced on average 43% of the seeds compared to control plants, and hermaphrodites produced 63% of the seeds compared to female plants (Table 2; Fig. 3A).

**Table 2:** Statistical results from the final Hurdle models analyzing seed production in 2002 and 2003.

Seed production 2002 (AIC = 731.7)				
*Count model*	Estimate	SE	Z value	P value
Intercept	4.529	0.191	23.71	<0.001
Shade	-0.560	0.220	-2.548	0.011
Hermaphrodite	-0.452	0.221	-2.039	0.041
Shoots (centered)	0.248	0.076	3.260	0.001
*Zero Hurdle model*	**Estimate**	**SE**	**Z value**	**P value**
Intercept	1.386	0.280	4.96	<0.001
**Seed production 2003** (AIC = 713.5)				
*Count model*	**Estimate**	**SE**	**Z value**	**P value**
Intercept	4.774	0.112	42.525	<0.001
*Zero Hurdle model*	**Estimate**	**SE**	**Z value**	**P value**
Intercept	0.712	0.239	2.974	0.003

**Fig 4:**
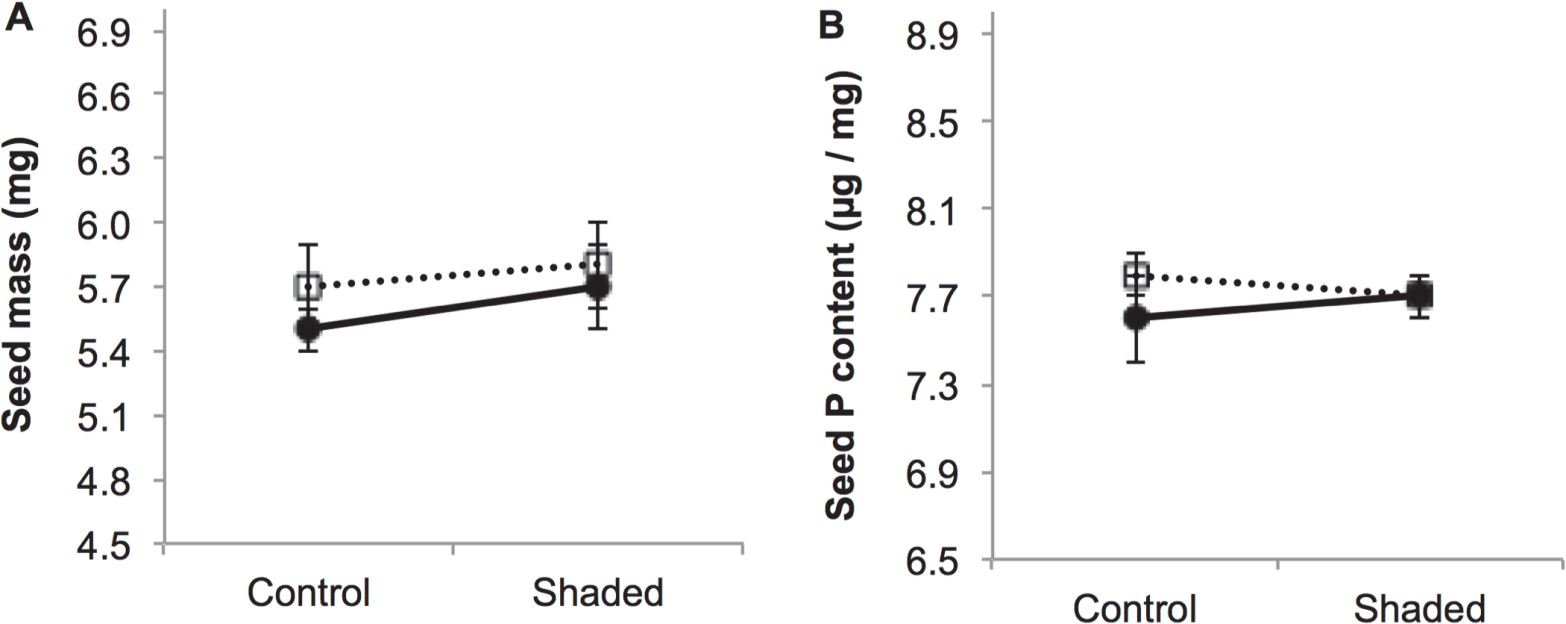
(A) Seed mass (in mg) and (B) seed P content (in μg P / mg seed) in in female (●) and hermaphrodite (□) control and shaded *Geranium sylvaticum* plants. Mean ± 1SE are indicated (N = 62 plants and N = 40 plants for (A) and (B) respectively).

**Fig 5:**
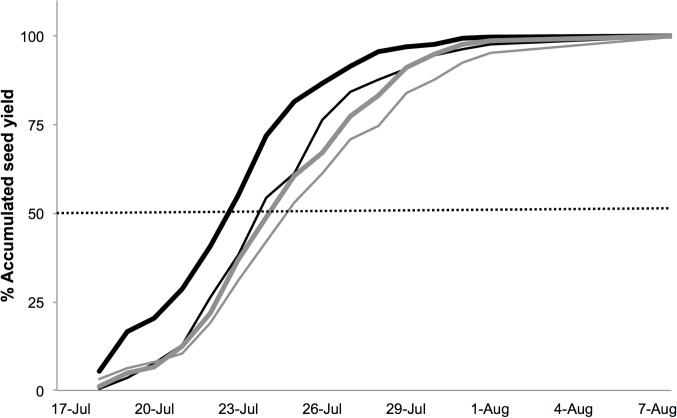
Percentage of accumulated seed yield produced per plant through the flowering season in 2002 in female (black lines) and hermaphrodites (grey lines) *Geranium sylvaticum* plants in control (thick lines) and shaded conditions (thin lines). The dashed horizontal line indicates 50% accumulated seed production.

Seeds had an average mass of 5.7 ± 0.1 mg and a similar P content regardless of any of the factors considered (Table 3; Fig. 4A, B). Variation in seed mass was 14% (range 3.5–7.6 mg) and mostly detected between individuals: within individuals, seed mass varied on average only by 7% and the seeds produced early in the season were not larger than the seeds produced later in the season (data not shown). Strong sexual differences in the process of seed production through the season and in response to shade were not observed (Fig. 5). Even though females growing in control conditions achieved 50% seed production three days earlier than control hermaphrodites and shading the plants delayed seed production in both sexes by two days (Fig. 5), these differences were not statistically significant (F_1,51_ = 2.99, P = 0.09; F_1,52_ = 1.69, P = 0.20, and F_1,50_ = 0.82, P = 0.37, for the effect of sex, shading, and the interaction between these two factors respectively).

**Table 3:** Statistical results from the analysis of variance for seed mass and seed phosphorus content.

	Seed mass			
	df	MS	F	P
Light	1	0.401	0.401	0.945
Sex	1	0.357	0.840	0.363
Light x Sex	1	0.131	0.309	0.580
Shoots (centered)	1	0.008	0.018	0.894
Residuals	56	0.425		
**Seed P**				
Light	**df**	**MS**	**F**	**P**
Sex	1	0.004	0.004	0.872
Light x Sex	1	0.070	0.070	0.524
Shoots (centered)	1	0.041	0.041	0.626
Residuals	1	0.004	0.004	0.876
	34	5.707		

The reduction in light availability imposed during the flowering season in 2002 did not affect plant survival or the probability of reflowering the following year. Only one experimental plant died between 2002 and 2003 (one control hermaphrodite) and 81% of the plants reflowered regardless of their sex or the shade treatment imposed the previous year (all P > 0.24). In 2003, 26 plants failed to produce any seed and this was not related to the sex or the light treatment (Table 2, Fig. 3B). Fruiting plants produced on average 118.3 ± 1.2 seeds irrespectively of the sex or the light treatment (Table 2; Fig. 3B).

## Discussion

The aim of this study was to investigate the existence of sex-differential plasticity in response to light availability during seed maturation using the gynodioecious plant *Geranium sylvaticum*. To test for SDP, we predicted reduced reproductive output in response to shade in hermaphrodites whilst females should keep a relatively similar reproductive output regardless of the experimental conditions. We found no evidence to confirm that *G*. *sylvaticum* exhibits SDP in our experimental population in response to light availability during seed maturation because reproductive output was reduced to similar degree in both sexes (i.e. no sex-by-treatment interaction). It should be pointed out that SDP does not necessarily require no reproductive output reduction in females, just that any reductions are less than that of hermaphrodites so that females have advantage (or greater advantage) in one environment, but none (or much less) in another.

The SDP hypothesis has been corroborated in several field studies showing a negative relationship between female frequency and resource availability (reviewed in [12], [14]). In addition to these field observations, there is some support for the SDP hypothesis gained with experiments manipulating resource availability ([11], [29], [30], [31], [32]; but see [16], [27], [23] and [33]). These studies strongly suggest a direct link between sex ratio and nutrient or water availability (reviewed in [15]), but there is evidence that this is not the case of light availability. In a greenhouse experiment, van Etten *et al*. [16] did not find support for SDP in response to light in *Geranium maculatum* and no differences in seed production between the sexes were detected. Later, the relationship between light availability and the distribution of the sexes was investigated in the field by van Etten and Chang [17] who concluded that SDP due to different light and soil moisture conditions were not the mechanism explaining the distribution of sexes. Similarly, in the present study seed reduction due to shade was similar in both sexes. Remarkably, differences in fecundity in *G*. *sylvaticum* in response to different resource availability have been shown previously ([23], [27], [30], [33]). Asikainen and Mutikainen [30] manipulated resource availability by applying fertilizer and showed that the reduction in seed production between plants growing in non-fertilized (thus more nutrient limited) compared to fertilized plants was almost double in hermaphrodites vs. females (43% vs. 28% as calculated from open-pollinated plants from Fig. 2 in [30]). In the experiments by Varga and Kytöviita ([23], [33]), there was no difference in seed production between the sexes in response to mycorrhiza. Mycorrhizal symbiosis increases plant nutrient acquisition and is used instead of fertilizers in organic farming. In another experiment, Varga *et al*. [27] defoliated plants and showed that defoliation decreased seed production in a similar manner in both sexes (51% in hermaphrodites and 45% in females). Defoliation reduces plant nutrient capital as well as its carbon acquisition capacity and according to SDP, hermaphrodite individuals should be more responsive than females to these. Taken all the four available studies together, it seems that SDP may take place in *G*. *sylvaticum*, but the response is far from ubiquitous. The response by the sexes may vary according to the type of resource limitation (different nutrients, carbon acquisition or both) and to the time of the resource limitation. Moreover, populations may also vary in their level of plasticity differences [32].

A basic assumption in life history theory in perennial plants is that there are trade-offs between resource allocation to reproduction and to other functions related to fitness [34]. The cost of reproduction can be manifested as increased mortality, reduced growth or reproductive effort the following year. In *G*. *sylvaticum*, costs of reproduction have been identified and have been reported to differ between the sexes. Seed production has been associated with a cost in terms of reduced flower production in hermaphrodites [18], [35] whilst in females supplemental hand pollination did not affect the number of flowers produced, but decreased seed production the following year [35]. Here we show that low light availability during seed maturation reduces seed production, but does not affect flowering or seed production the following year. We expected to find a correlation in seed production between the two years as a result of trade-offs and costs of reproduction. However, fecundity may vary between years in *G*. *sylvaticum* ([24], this study) and since we only measured survival and reproductive output in two consecutive years, we cannot exclude that the costs of reproduction were present as a reduced growth during the year following light treatment or as a reduced allocation to roots.

Because seeds were collected daily it was possible to compare sexual differences in the process of seed production through the season and in response to shade. Shading during seed maturation decreased the total number of seeds produced, but did not affect seed production rate, seed mass or P content. Females growing under open sky finished fruiting about three days earlier than hermaphrodites but produced seeds of similar quality (size, P content). We did not note when the *G*. *sylvaticum* individuals started flowering, but slight differences in flowering phenology between the sexes have been reported, with females starting flowering three days earlier than hermaphrodites, even though variation among populations exists [36]. Therefore, it seems logical to assume that this difference in the onset of flowering is translated later on in seed production. Moreover, shading the plants delayed seed production in both sexes by two days, although this was not statistically significant. Shade has been reported to modify not only flowering phenology, but also seed production in other species including *Geranium maculatum* [18]. Probably, the subtle effect of shading on seed production rate in our study was due to the experimental setup, because shading was applied after most of the flowers were already produced. Compared to seed size, seed nutrient contents in gynodioecious plants are rarely measured and as far as we are aware of, all available reports show no sexual differences in seed nutrient contents [37], [38], [39], including the present study. It is unclear how light availability affects seed nutrient provisioning even though day length and light quality during seed development may affect seed germination through effects on the phytochrome (see e.g. [40] and references therein). Altogether, our results indicate that females and hermaphrodites responded in a similar way to shade.

It should be kept in mind that while our experimental design allowed us to study the plants in their natural environment, potential confounding effects might have been overlooked if the sexes occupy different microhabitats or if shading had an effect not on the plants themselves, but on the environment around the plants. For example, significant spatial population structure due to sex was demonstrated for the close species *G*. *maculatum* [17], where females were located in significantly brighter microsites than hermaphrodites. We cannot rule out this possibility for our species, but because we chose pairs of plants in blocks randomly within the population and shaded half of them, the potential differences in microsites should be minimal. An increased shade could have had also indirect effects through affecting foraging of pollinators. As reported in other plants [41] and also in the close species *G*. *maculatum* [42], plants may receive less pollen as either the density of the tree canopy or the cloud cover increases in a forest with no shrub layer. Changes in light availability may have sex-specific effects on pollination success through affecting the thermal biology of pollinators or the stigma receptivity and pollen germination rates [43]. However, we placed the shading frames after most of the flowers were pollinated and therefore the effect on pollinators could be assumed to be negligible and the differences in seed production should not be due to pollen availability.

In conclusion, this study demonstrates the absence of reproductive sex differential plasticity in response to light availability during seed maturation in the present population of the gynodioecious species *Geranium sylvaticum*. Given that differences in plasticity among populations may exist [32], studies including more populations are needed to generalize these results. Available evidence suggests that other factors such as pollen limitation, selfing and inbreeding depression and biotic interactions may act jointly to explain the maintenance of females in this gynodioecious species.

## Supporting Information

S1 Table Statistical results from the full Hurdle models analyzing seed production in 2002 and 2003. (DOCX)
