# Supplementary material for: Absence of Sex Differential Plasticity to Light Availability during Seed Maturation in Geranium sylvaticum
Source: PLoS One. 2015 Mar 4;10(3):e0118981. doi: 10.1371/journal.pone.0118981 (PMC4349700; doi:10.1371/journal.pone.0118981)
Supplement: S1 Table — (DOCX) [file pone.0118981.s001.docx]

**S1 Table. Statistical results from the full Hurdle models analyzing seed production in 2002 and 2003.**

| **Seed production 2002** (AIC=737.6) | | | | |  | **Seed production 2003** (AIC=721.9) | | | | |
| --- | --- | --- | --- | --- | --- | --- | --- | --- | --- | --- |
| *Count model* | **Estimate** | **SE** | **Z value** | **P value** |  | *Count model* | **Estimate** | **SE** | **Z value** | **P value** |
| Intercept | 4.422 | 0.208 | 21.291 | <0.001 |  | Intercept | 0.729 | 0.218 | 21.658 | <0.001 |
| Shade | -0.349 | 0.299 | -1.168 | 0.243 |  | Shade | 0.076 | 0.338 | 0.225 | 0.822 |
| Hermaphrodite | -0.247 | 0.295 | -0.837 | 0.402 |  | Hermaphrodite | 0.046 | 0.309 | 0.150 | 0.881 |
| Shade x Hermaphrodite | -0.445 | 0.435 | -1.030 | 0.303 |  | Shade x Hermaphrodite | -0.061 | 0.454 | -0.134 | 0.894 |
| Shoots (centered) | 0.246 | 0.076 | 3.252 | 0.001 |  |  |  |  |  |  |
| *Zero Hurdle model* | **Estimate** | **SE** | **Z value** | **P value** |  | *Zero Hurdle model* | **Estimate** | **SE** | **Z value** | **P value** |
| Intercept | 1.708 | 0.630 | 2.713 | 0.007 |  | Intercept | 0.847 | 0.488 | 1.736 | 0.083 |
| Shade | -0.215 | 0.857 | -0.250 | 0.802 |  | Shade | -0.847 | 0.662 | -1.280 | 0.201 |
| Hermaphrodite | -0.027 | 0.890 | -0.031 | 0.976 |  | Hermaphrodite | 0.182 | 0.714 | 0.255 | 0.798 |
| Shade x Hermaphrodite | -0.559 | 1.165 | -0.480 | 0.632 |  | Shade x Hermaphrodite | 0.916 | 0.988 | 0.927 | 0.354 |
| Shoots (centered) | -0.189 | 0.167 | -1.131 | 0.258 |  |  |  |  |  |  |
